# Health systems readiness for integration of point-of-care digital diagnostic tools for acute febrile illness in Ghana: a mixed-methods study protocol

**DOI:** 10.1136/bmjph-2025-003704

**Published:** 2026-07-14

**Authors:** Shola Molemodile Dele-Olowu, Leonard Baatiema, Frances Baaba da-Costa Vroom, Emilia A Udofia, Luc de Witte, Alfred Edwin Yawson, Julie Balen, on behalf of the NIHR Global Health Research Group on Digital Diagnostics for African Health Systems

**Affiliations:** 1 School of Public Health, University of Ghana, Accra, Ghana; 2 University of Ghana Medical School, Accra, Ghana; 3 The Hague University of Applied Sciences, The Hague, The Netherlands; 4 Community Health, University of Ghana Medical School, Accra, Ghana; 5 Canterbury Christ Church University School of Allied and Public Health Professions, Canterbury, UK

**Keywords:** Digital Technology, Public Health, Communicable Disease Control

## Abstract

**Introduction:**

The convergence of digital technologies with point-of-care (POC) diagnostics has paved the way for innovative digital POC diagnostics that combine the convenience and accessibility of POC testing with digital connectivity and data analytics capabilities. Given the burden and mortality from infectious diseases such as malaria in many African countries, rapid diagnosis and treatment are essential for effective disease management. Therefore, this study aims to assess the readiness of African health systems, with a focus on Ghana, to integrate new POC digital diagnostic tools for acute febrile illness by directly learning from stakeholders and intended users.

**Methods and analysis:**

This study focuses on Ghana’s health system within a broader context of work across six sub-Saharan African countries. It will adopt a mixed-methods approach to assess the readiness of Ghana’s health system to use and integrate new POC digital diagnostic tests for diagnosing acute febrile illness.

Data will be collected through a realist evaluation approach, policy analysis, surveys (n≈75), key informant interviews (n≥20), focus group discussions and observations in three regions in Ghana.

Stakeholders representing various levels of health system governance, including global, national, regional and district levels, will be purposively selected for qualitative interviews. Analysis will be guided by realist evaluation using context–mechanism–outcome configurations, complemented by a four-by-four implementation framework. Qualitative data will undergo thematic analysis, while quantitative survey data will be analysed using descriptive and inferential statistics. Findings will be triangulated across data sources to refine programme theory and identify context-specific pathways for integration.

**Ethics and dissemination:**

The study aims to contribute to the design of new POC digital diagnostic tests that are context-appropriate for diagnostic testing in Ghana and similar African contexts. Results will be communicated to relevant communities and stakeholders and disseminated through publications and presentations.

**PROSPERO registration number:**

CRD420251084372.

**WHAT IS ALREADY KNOWN ON THIS TOPIC**

- Digital point-of-care (POC) diagnostics have the potential to expand access to timely, accurate diagnoses in low- and middle-income countries, especially for infectious diseases. Yet simply introducing diagnostic technologies without a system ready to integrate them is insufficient to improve health outcomes.

**WHAT THIS STUDY ADDS**

- This study applies a realist evaluation approach combined with a four-by-four implementation framework to examine how, why and under what circumstances digital POC diagnostics can be integrated into Ghana’s health system. It provides context-specific insights from key stakeholders into mechanisms that influence health system readiness for integration and develops an empirically grounded programme theory to guide implementation in similar contexts.

**HOW THIS STUDY MIGHT AFFECT RESEARCH, PRACTICE OR POLICY**

- Findings will guide design and implementation of context-appropriate digital diagnostic interventions by identifying key health system requirements and integration pathways. More broadly, the study contributes to implementation science by advancing methodological approaches for assessing health system readiness in low-resource settings.

## Introduction

 Point-of-care (POC) digital diagnostic tools represent a transformative shift in healthcare delivery by reducing the turnaround time for test results and expanding access to essential diagnostics. These tools are particularly critical in low- and middle-income countries (LMICs), where access to diagnostics on the WHO essential list remains limited due to financial barriers and geographical distance from centralised laboratories.^1^,^3^ In sub-Saharan Africa, the burden of infectious diseases, including malaria and other febrile illnesses, underscores the urgent need for timely, accurate and decentralised diagnostic capabilities.^4^,^7^

A health system’s readiness to integrate new technologies is an essential consideration when implementing digital POC tools. Readiness refers to the preparedness of healthcare systems, providers and patients to use POC diagnostic devices effectively, including provision of required infrastructure, resources, training and monitoring compliance.^8^ Integration involves incorporating POC diagnostics into existing healthcare workflows and information systems, enabling efficient data exchange and the incorporation of results into decision-making.^9^

The spectrum of POC diagnostic technologies continues to evolve with advancements in digital health and medical technologies. These include portable handheld devices, smartphone-based diagnostics, wearable tools and lab-on-chip devices.^10^ These innovations are particularly useful in resource-limited settings and remote areas where access to traditional laboratory facilities is limited, enabling rapid testing and analysis of biological samples, such as blood, saliva or urine, in a portable and compact format.^811^,^14^ Rapid test results from POC diagnostics enable healthcare providers to make informed treatment decisions in a timely manner.^15 16^

Digital POC diagnostics combine traditional testing methods with the benefits of digital connectivity, enabling remote data access, real-time analytics and integration with health information systems.^17 18^ However, large-scale, context-specific evidence to guide the effective and sustainable integration of these tools into African health systems remains limited. While general frameworks for scaling digital health solutions exist, studies documenting widespread deployment and system-level integration are scarce.^19^ In Ghana, for example, where this study is situated, the national rollout of POC tests for syphilis did not significantly increase antenatal testing rates.^20^ Likewise, POC testing for maternal health was both unavailable and inaccessible in rural primary healthcare (PHC) facilities.^21 22^

Rapid diagnostic tests (RDTs) for malaria have proven essential and exemplify the dual promise and complexity of implementing POC diagnostics. While they are crucial in resource-constrained settings lacking advanced laboratory infrastructure, challenges persist.^23^ These include product variability in sensitivity and specificity, susceptibility to environmental extremes and logistical barriers such as irregular supply chains and inadequate storage.^24^ Evidence from Ghana and Nigeria suggests that the effectiveness of malaria RDT deployment is undermined by recurrent stockouts, inconsistent supply chains and a persistent reliance on presumptive treatment despite national test-and-treat policies.^25^,^27^ Moreover, sociocultural and health system dynamics, including clinician trust in diagnostics, turnover among trained personnel, regulatory barriers, funding limitations and quality assurance gaps, limit sustained use and mainstreaming of digital diagnostics.^1721 28^,^36^ The Lancet Commission on Diagnostics further emphasises that the lack of visibility and priority for diagnostics in many LMICs is the root cause of limited access to essential diagnostic tests.^3^

Overall, this study aims to assess Ghana’s health system’s readiness to integrate and use new digital diagnostic tests for febrile illness. It will explore policy perspectives and health system requirements for integrating context-appropriate POC digital diagnostic tools in African health systems.

## Methods and analysis

This study aims to understand the national policy and governance environments surrounding the integration of digital diagnostics into African health systems, using Ghana as a case study. It will also aim to answer the questions of how and under what circumstances a new POC digital diagnostic tool for the diagnosis of acute febrile illness, such as malaria, can be integrated into Ghana’s health system.

The study aims to answer the following research questions:

1. What is Ghana’s existing policy and health system landscape for digital diagnosis of infectious diseases?
2. What are the perceptions, expectations, needs and concerns of various health system stakeholders regarding the integration of a new POC digital diagnostic for febrile illness in Ghana?
3. How may contextual factors impact the readiness for integration and sustainability of a new POC lab-on-chip technology for diagnosing malaria in Ghana’s health system?

Specific objectives for this study are to:

1. Understand the policy and health system landscape for digital diagnosis of infectious diseases in Ghana.
2. Explore multistakeholder (policymakers and implementers across different levels) perceptions, expectations, needs and concerns about integrating a new POC digital diagnostic for febrile illness in Ghana.
3. Understand factors that impact the readiness for integration and sustainability of a new POC lab-on-chip technology for diagnosing malaria in Ghana’s health system.

### Theoretical framework

The study adopts a multiframework approach to analyse the integration of digital diagnostic tests in African healthcare settings. The frameworks adapted are (1) the realist evaluation (RE) method^37^ and (2) the four-by-four framework for the adoption and scale-up of interventions.^38^ Together, these frameworks enable an in-depth exploration of how context, mechanisms, outcomes and health system readiness influence the implementation and scaling of digital diagnostic tools.

**Figure 1:**
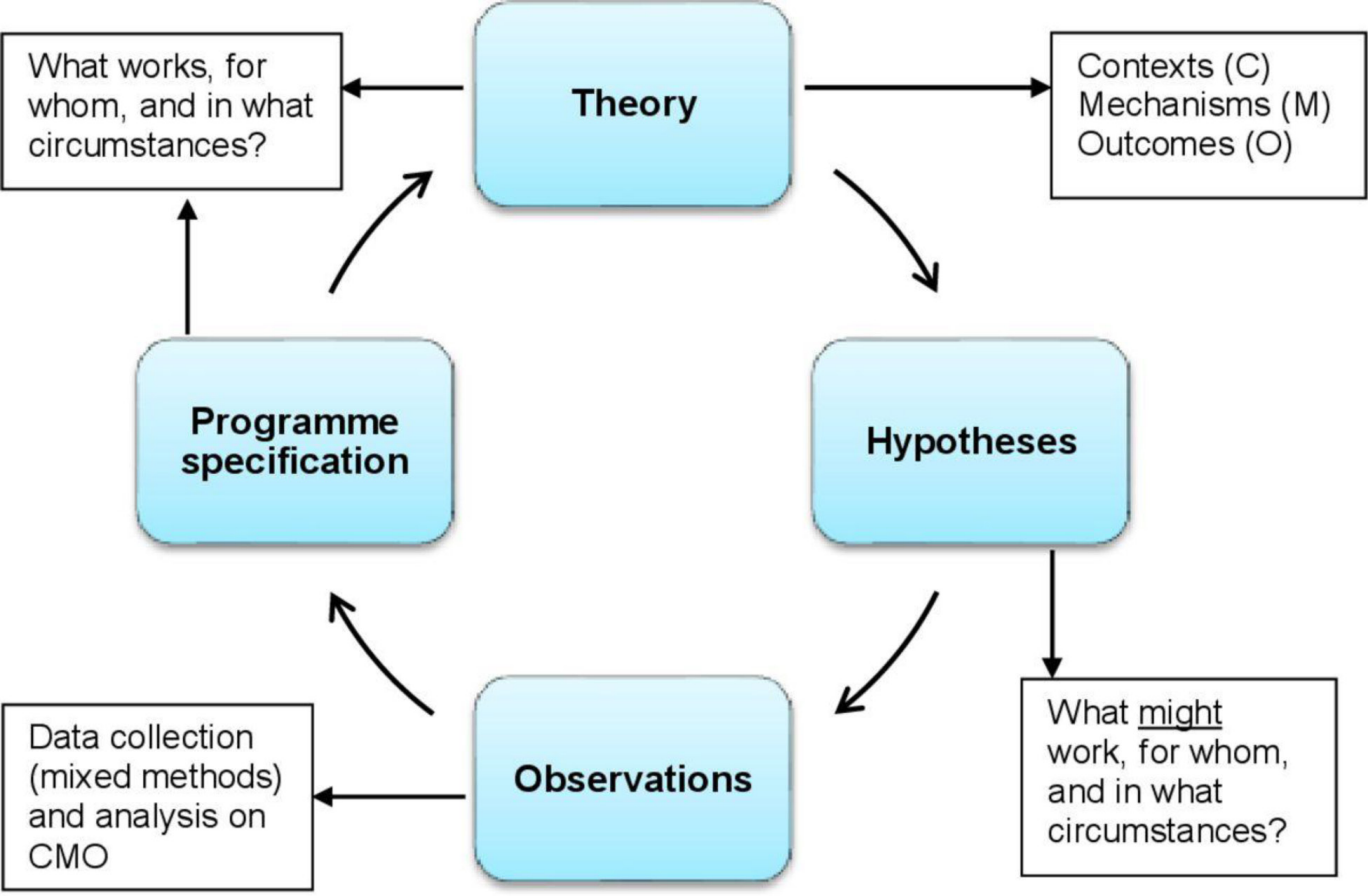
The realist evaluation cycle (Pawson and Tilley).

The RE approach is a theory-driven method for studying complex health system interventions.^39^ It examines the causal relationships between contexts (C), mechanisms (M) and outcomes (O) that are likely to be important given the intervention and the outcome(s) of interest. This approach is known as a C-M-O configuration (figure 1), which accounts for multiple dimensions and documents all key aspects of an intervention, thereby contributing to the validity and reliability of results.

The RE approach, rooted in social theory, is increasingly employed in global health research, as it provides a structured way to assess implementation feasibility and effectiveness and to address questions about what works, in which setting, for whom, under what circumstances and why. In this case, the involvement of policymakers and stakeholders as potential end-users of a new POC digital diagnostics (intervention) is central to the integration process. Broader community/stakeholder engagements (mechanism) will be required for POC digital diagnostic integration (outcome) in Ghana’s health system to ensure the intervention research is context-fit and approved by local communities.

**Figure 2:**
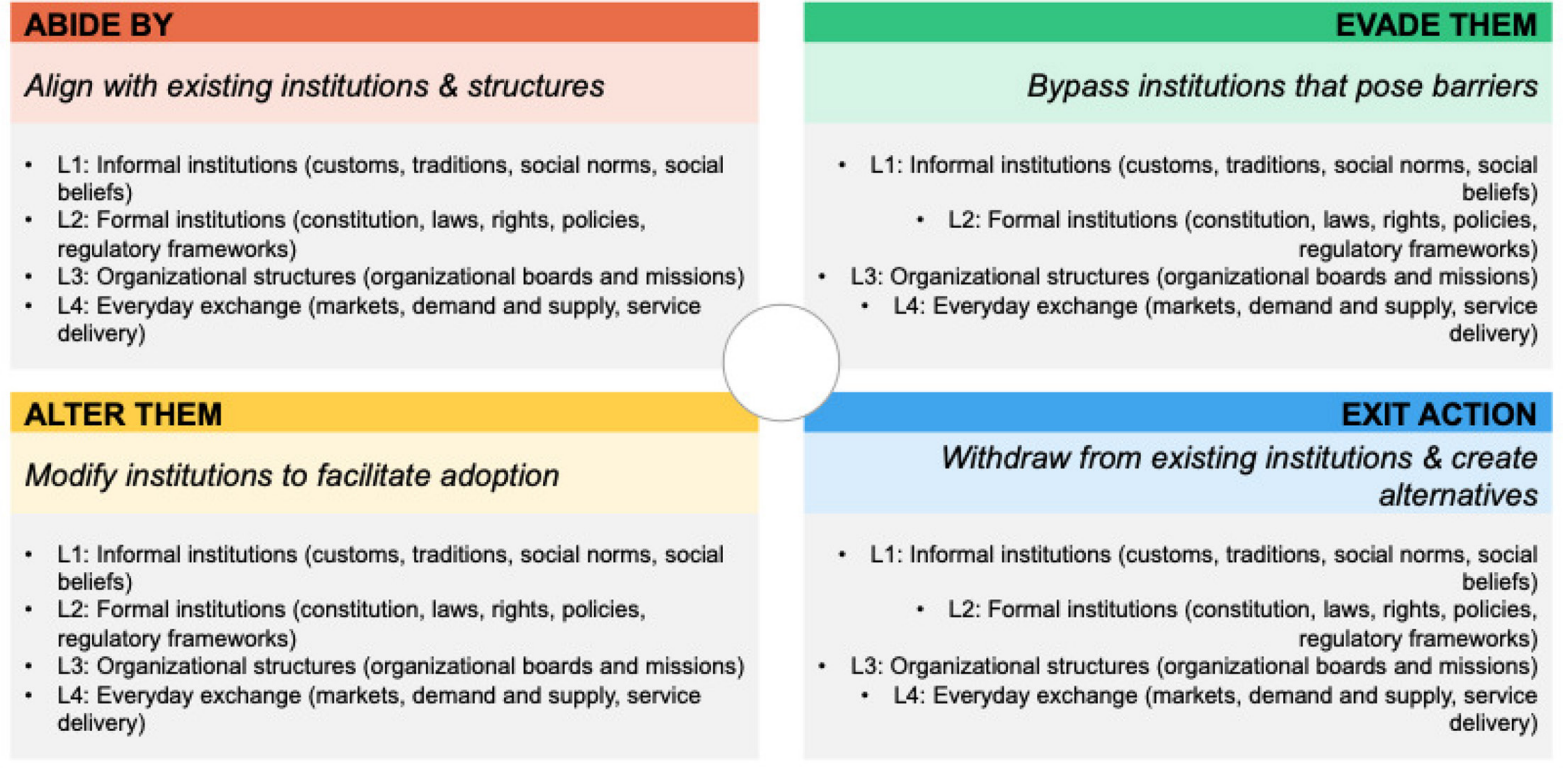
Four-by-four framework for adoption and scale-up of interventions.

To complement the RE and explore the broader process of intervention adoption, the study will apply the four-by-four framework for the adoption and scale-up of interventions, as proposed by Abimbola and Liu.^38^ This framework (figure 2) categorises implementation processes across four institutional dimensions: informal institutions, formal institutions, organisational structures and everyday exchanges. This is done across four stages of implementation strategies: abide by institutions, evade them, alter them and exit action.

By mapping the data across these four dimensions and implementation strategies, we aim to contextualise the RE findings within a broader framework that accounts for both the microlevel and macrolevel factors influencing the integration of digital diagnostic tools.

### Study design

This mixed-methods study examines the readiness of Ghana’s health system to integrate and use a new POC digital diagnostic tool at service delivery points. An initial programme theory, following a comprehensive review of the literature and existing policy documents, will be developed to account for how the *context* in which interventions are intended influences the *mechanisms* (eg, roles of policy and community stakeholders in the design and implementation of digital diagnostic interventions) to yield *outcomes*, both intended and unintended. This context–mechanism–outcome (CMO) configuration will be continuously validated and refined throughout data collection and analysis. Qualitative and quantitative methods will be employed to collect and analyse data, drawing insights into whether and how implementing a new POC digital diagnostic tool is feasible across different governance levels national, regional, district and community in Ghana’s health system. The focus of this study is on Ghana. Still, to better understand how global and regional policies translate into the country, we would also conduct a survey of African health systems and diagnostic experts to gather information on health system readiness for integrating new POC digital diagnostics across the continent. This mixed-methods health systems research will combine surveys of policymakers across African countries and an in-depth case study in Ghana, using evidence synthesis, document review, quantitative data, in-depth interviews and group discussions to answer the research questions.

### Study setting

Ghana is located north of the Guinea Coast in West Africa, bordered by Burkina Faso to the north, Togo to the east and Côte d’Ivoire to the west. With an estimated population of 35 million in 2025, the country comprises 16 administrative regions and a local government system comprising 261 administrative and political districts, categorised into metropolitan, municipal and district assemblies, which have limited legislative powers.^40^,^42^ Ghana is also divided into three ecological zones: the northern savannah, the middle forest and the southern coastal savannah belts, each exhibiting significant differences in climate, demographics and socio-economic characteristics. Malaria, along with lower respiratory tract infections, neonatal infections, ischaemic heart disease, strokes, HIV/AIDS and tuberculosis, has been a leading cause of death in the country.^43^

With oversight from the ministry of health, the country provides healthcare through a pluralistic health system that includes both the public and private sectors, as well as traditional and complementary medicine. Healthcare services operate through a three-tier system: primary (district), secondary (regional) and tertiary (national) levels. District health services are organised into three levels: community-based health planning and services (CHPS zones), subdistricts (health centres and clinics) and districts (district hospitals and district health directorates). The Ghana Health Service (GHS), the public agency responsible for PHC, features a deconcentrated structure comprising ten national divisions, 16 regional entities and 216 district health directorates.

### Sampling and study population (inclusion and exclusion criteria)

A multistage stratified sampling approach will be employed to assess Ghana’s health system readiness for effectively integrating a POC digital diagnostic tool. This method will ensure a representative and comprehensive selection of stakeholders across different health system levels while accounting for variations in geography, infrastructure and facility ownership.

The first stage of sampling will involve selecting areas across Ghana’s 16 administrative regions. These regions differ in health system capacity, economic development and urban–rural distribution, necessitating stratification based on geographical and ecological diversity, resource availability and disease burden. The selection will ensure representation from Ghana’s Northern, Middle and Southern zones, capturing regions with high, medium and low health system capacities while balancing metropolitan and rural settings.

After regional selection, the second stage will focus on stratifying districts within the selected areas. Districts will be chosen based on their capacity for health service delivery, socio-economic status and urban-rural mix. This selection will ensure inclusion of districts with secondary-level facilities (regional hospitals) as well as primary-level and community health centres representing various levels of healthcare provision. The study will address healthcare access and infrastructure disparities by incorporating wealthier and lower-income districts.

The third stage will involve sampling health facilities within selected districts to ensure diversity in facility type and ownership. Public tertiary, regional and district hospitals, as well as private hospitals, clinics and PHC centres (including CHPS compounds), will be included. Facilities will be further categorised by their readiness for POC diagnostic integration, ranging from well-resourced facilities with laboratories and digital infrastructure to lower-resource centres with limited diagnostic services. This approach will ensure a comprehensive system readiness assessment at service delivery level.

To complement facility-based sampling, purposive sampling will be used to recruit key informants across different levels of the health system. At national level, representatives from the ministry of health, GHS, regulatory agencies and development partners will be engaged. Health directors and public and private sector leaders will be included at regional and district levels. Facility-level stakeholders such as medical superintendents, diagnostic service providers and frontline healthcare workers will provide critical insights into operational feasibility, challenges and opportunities for POC diagnostic implementation. These key informants will be knowledgeable adults who have experience with or play key roles in introducing and using diagnostics in the study area. Exclusion criteria include stakeholders with less than 2 years of experience and those not directly involved in health service delivery or policy.

To maintain rigour in data collection, sampling will continue until thematic saturation is reached in qualitative interviews. This comprehensive sampling strategy will enable a holistic, contextually relevant evaluation of Ghana’s health system’s readiness to integrate POC digital diagnostic tools.

In this study, calculating a strict sample size may not be necessary because of the following:

- The exploratory and qualitative nature of the study focuses on understanding processes, barriers, facilitators and stakeholder perspectives rather than measuring precise numerical outcomes related to health system readiness.
- Stakeholder-driven approach: The selection of participants (eg, policymakers, healthcare managers and frontline workers) is purposive, aiming for diverse representation rather than statistical generalisation.
- Thematic saturation: The qualitative component relies on data saturation, meaning interviews will continue until no new insights emerge rather than reaching a predefined sample size.
- Facility-level data: While a broad range of health facilities will be included, the focus is on representative diversity (public vs private, urban vs rural, different service levels) rather than achieving a statistically powered sample.

For objective 1, study populations will include policymakers and regulators, health system administrators, private sector technology providers/manufacturers and health systems experts.

For objective 2, in addition to the stakeholders listed above, policy implementers from different levels of the health system, including programme managers and health workers, that is, clinicians, laboratory technicians, scientists, community health workers from both public and private sectors and non-governmental organisations, will be included in the study population.

For Objective 3, all study populations will be included.

### Recruitment of participants

Recruitment will be conducted based on a mix of purposive and snowball sampling methods to ensure a diverse representation of stakeholders. An initial stakeholder mapping exercise will identify key informants from across health system governance levels, that is, global, national, regional and district health levels. Recruitment will then involve direct invitations facilitated by a gatekeeper (eg, academic advisors, senior leadership and management staff at GHS). Each key informant interviewed will be asked for prospective interviewees, and a list will be compiled for either interviews or a stakeholder meeting. In this way, a range of stakeholders will be identified for either interviews or a stakeholder meeting once thematic saturation occurs during interviews. One stakeholder meeting is proposed during this research’s formative and summative phases in Q1 2023 and Q2 2026, respectively. The first stakeholder meeting will aim to introduce the study and engage with initial stakeholders, and the second will share findings and engage policymakers cocreatively on possible use of the results.

Recruitment for focus groups and health facility surveys will involve contacting healthcare workers across different levels of care (community, district and regional hospitals). This will be coordinated through existing professional networks and regional health offices.

### Data collection

#### Phase I: initial theory development

Literature and publicly available documents on digital health and POC diagnostics across Africa will be reviewed. Informal discussions with three to five key stakeholders across different health governance levels (ie, national, regional and district levels) in Ghana will also be held to guide theory development and refinement. In addition, available evidence on existing frameworks to guide integration of digital diagnostics into health systems will be reviewed (*Phase I aims to meet objectives 1 and 2*). Study tools will be developed to guide formal key informant interviews and focus group discussions. Tools will be pilot-tested, and topic guides will be iteratively adapted during data collection to refine questions and identify new areas of inquiry as needed. A systematic review will examine factors influencing health system readiness and integration of existing tools, such as accessibility, affordability, infrastructure requirements and healthcare provider training in sub-Saharan Africa. Data collection is planned for late 2025 to mid 2026. Evidence will be summarised on the gaps and factors affecting readiness, integration and use of existing tools within health systems.

#### Phase II: validation and refining of theory

*Qualitative data:* Collection tools will include in-depth interviews, focus group discussions and non-participant observations. Interview guides will be developed based on the initial theory development phase and refined iteratively (see online supplemental file 2). Data will be collected using digital recorders and transcribed for analysis. Qualitative data will be obtained from at least 20 in-depth interviews (at national level and across the three regions), six focus group discussions (split across the regions among three groups, ie, end users/healthcare providers, laboratory personnel per region) and non-participant observations (at one CHPS compound, one health centre, one district hospital and one regional hospital). Collated information will be organised into themes, subthemes and verbatim quotes to validate the theories elicited from literature and policy documents. Participants for interviews and focus groups will be identified through a ‘gatekeeper’, namely the senior leadership of the various stakeholder institutions, including GHS. Participants will be contacted via email and/or phone calls. The researcher will schedule in-person interviews with each potential participant who has indicated interest. Written informed consent will be obtained before the interview begins. Key informant interviews with policymakers on experience with introducing and ensuring the sustainability of digital diagnostic tools will be conducted (*objectives 1, 2 and 3*). To address objective 3b, participatory methods will be employed, engaging policy leaders in discussions to identify context-fit approaches for introducing new digital diagnostic devices into Ghana’s health system. This primary qualitative data collection will be supplemented by using policy documents at different levels of government.

*Quantitative data:* To capture perceptions on integration and feasibility of digital diagnostics, surveys will be administered to healthcare workers and policymakers across different healthcare system levels. The questionnaire will capture data on the use of POC diagnostics, perceptions of digital diagnostic tools and barriers to integration. Data collection will be facilitated using tablets with preloaded digital survey software (Research Electronic Data Capture (REDCap)). A targeted questionnaire will be administered to clinicians, laboratory managers and other health workers involved in testing and diagnosis of infectious diseases across selected health facilities across all levels of the healthcare system, namely primary (district hospitals), secondary (secondary health facilities) and tertiary healthcare facilities (teaching hospitals) from across purposively selected regions of Ghana. The survey instrument will be developed using the initial programme theory and recognised, validated techniques for assessing health system readiness and digital health adoption. It will be pilot tested with five to seven healthcare workers and policymakers from non-study sites to assess clarity, feasibility and completion time. Feedback will guide improvements to wording, response choices and overall structure. The validity and reliability of survey tools will be thoroughly tested. Face validity will be determined by a review by a group of three to five specialists in health systems, digital health and diagnostics. Content validity will be ensured by mapping survey items against WHO health system building blocks and established readiness components to achieve broad and comprehensive coverage. Furthermore, test–retest reliability will be assessed with a subset of pilot participants over a 2-week interval to determine the stability of responses over time. Survey tools are included as online supplemental file 3.

This will elicit perspectives on organisational readiness and health system requirements, including training needs, supply chain, data processes and other relevant aspects, as well as experience with existing POC digital and non-digital diagnostic tools. Additionally, it will gauge the perception of a new digital diagnostic POC device for infectious diseases.

#### Phase III: consolidation of study findings and recommendations

Programme theories elicited will be summarised to articulate a rigorous model of the complex relationship between the context, mechanism and outcomes of introducing and sustaining POC digital diagnostics in Ghana’s health system. To consolidate findings and ensure adoption and integration, ideation workshops will be conducted to validate findings from the field and facilitate the evolution of ideas. Workshop outcomes will be reported to stakeholders.

### Data analysis

The data analysis will adopt an iterative and mixed-methods approach, beginning with a comprehensive literature and policy review to establish the theoretical and regulatory context for digital diagnostics. Qualitative and quantitative findings will be triangulated to assess Ghana’s readiness for integrating POC digital diagnostic tools. Key variables examined will include diagnostic guidelines, regulatory frameworks, leadership and governance structures, financing models and stakeholder perceptions regarding the availability, accessibility and effectiveness of existing digital diagnostics.

Qualitative data from interviews and focus groups will be thematically analysed and coded to identify CMO patterns using RE. These patterns will be categorised within a ‘four-by-four’ framework to analyse institutional and entrepreneurial strategies relevant to scaling digital diagnostics in complex health systems.

The analysis will involve continuous triangulation of the different data sets to extract outcomes. Two researchers will independently code a subset of transcripts to establish intercoder reliability, with discrepancies resolved through discussion. The researchers will engage in a stepwise process to ensure the credibility and dependability of findings, namely: (1) review interview notes and transcripts, comparing transcripts to audio tapes to ensure accuracy of transcription; and (2) input transcripts into a qualitative analysis software package that allows text to be numbered, coded and sorted. Results will be summarised in a report that includes relevant direct quotes. The researchers will be reflexive and document how their background might influence the results reported. A detailed description of all interactions will be provided to enhance the transferability of the results.

Quantitative survey data will be analysed using descriptive and inferential statistics in Stata (V.18) to summarise stakeholder perceptions, while inferential analysis will explore associations between contextual factors and observed outcomes. Descriptive analyses will summarise frequencies, proportions, medians and IQRs. Inferential analyses will examine associations between health system factors and readiness for integration. χ² tests will assess associations between categorical variables. Mann-Whitney U or Kruskal-Wallis tests will be used for non-parametric comparisons of ordinal readiness scores. Multivariable logistic regression will be conducted to identify predictors of readiness for integration (eg, infrastructure, training, governance factors), adjusting for facility and respondent characteristics.

### Data management

The study team will take all precautions to secure the data under lock and key. The data will be contained on a secure server behind University of Ghana’s firewall, only accessible through a password-protected computer by the research team. All printed copies will be stored securely in a locked cabinet in the research team’s office.

### Patient and public involvement statement

Patients will not be directly involved because the review focuses on health system readiness for digital diagnostic tools at the organisational and policy levels. Instead, relevant public stakeholders, including policy actors and frontline health workers, will be engaged. Stakeholders will be involved prospectively from the initial programme theory development stage and throughout the review to refine CMO configurations and ensure alignment with real-world implementation processes. Engagement will occur through targeted consultations (eg, workshops or structured feedback), with efforts to minimise burden through flexible participation and use of concise, plain-language materials. Stakeholders will also inform dissemination by co-interpreting findings and identifying appropriate policy and practice audiences.

## Ethics and dissemination

The study is registered in PROSPERO (registration number CRD420251084372). Reporting will follow the RAMESES II (Realist And MEta-narrative Evidence Syntheses: Evolving Standards) reporting standards for REs^39^ and the Good Reporting of A Mixed Methods Study framework (see online supplemental material 1).^44^ Quantitative components will also be reported in line with Strengthening the Reporting of Observational Studies in Epidemiology (STROBE) guidelines^45^ where applicable. Informed consent will be obtained, and participation in the study is voluntary, with the option to withdraw at any time. The benefit for participants in the study is that they can contribute and share their insights and perceptions, thereby informing improvements to the existing framework for integrating digital diagnostics in-country. All data collected for publications will be de-identified before use. The research team will take necessary precautions to ensure that any data obtained from the demographic survey is secure and kept confidential in a password-protected folder or locked cabinet. There are no anticipated risks to participants. No experimental procedure or interventions will be implemented during the study.

The study findings will be disseminated through multiple channels, including publications, conferences and workshops.

## Supplementary material

10.1136/bmjph-2025-003704 online supplemental file 1

10.1136/bmjph-2025-003704 online supplemental file 2

10.1136/bmjph-2025-003704 online supplemental file 3

10.1136/bmjph-2025-003704 online supplemental file 4

## Data Availability

No primary data are available as this is a study protocol. The study instruments are included as online supplemental files. Data generated from this study will be made available upon reasonable request after the study is completed.
