# Supplementary material for: Health systems readiness for integration of point-of-care digital diagnostic tools for acute febrile illness in Ghana: a mixed-methods study protocol
Source: BMJ Public Health. 2026 Jul 14;4(3):e003704. doi: 10.1136/bmjph-2025-003704 (PMC13374460; doi:10.1136/bmjph-2025-003704)
Supplement: online supplemental file 2 [file bmjph-4-3-s002.pdf]

Title: Assessing the readiness of Ghana's health system to integrate new digital diagnostic tools

Introduction

Thank you for participating in this key informant interview. Your expertise and perspectives are crucial to our efforts in assessing Ghana's readiness to integrate new digital diagnostic tools for infectious diseases.

Readiness is the ability of a health system to rapidly and sustainably adapt policies, processes and infrastructure to support the integration of a component of care.

Integration is the adoption and assimilation of a component of care into every aspect of a health system (i.e. governance, regulation, workforce, reimbursement and service delivery frameworks) to ensure its availability to all people who may benefit from it.

The information gathered will contribute to understanding the landscape and shaping policies and guidelines for successful integration. Your responses will remain confidential.

Understanding the context and roles of key stakeholders within the health system

1. Can you explain your role within the health system at (*name of organization and focus areas*)? Do you, or your organisation, have any role in diagnostics? How long have you been in this role? Any additional role or previous experience in diagnostics and/or digital diagnostics outside the current role?
2. What can you tell me about the current state of diagnostic testing in Ghana's health system (or the African health system where you work)? (*Probe in terms of infectious diseases or in general*). Has this changed over time? Please describe.

3. How would you describe the strengths of the current diagnostic system? What about its weaknesses? (*Probe for why these weaknesses exist*)
4. Are there any digital diagnostic tools readily available? If so, please provide some details. If not, why may that be the case? What are the pros and cons of these existing tools?

Governance and Leadership

5. Is there a global/regional/national policy/strategy/plan that appropriately includes digital diagnostics?
6. What specific policy frameworks or regulations currently exist to facilitate the integration and adoption of digital diagnostic tests within the healthcare system?
    a. Is there national leadership and political support for the integration of digital health broadly? Please describe.
    b. In your view, what are the key incentives provided by current health sector policies to encourage the development, adoption, and utilization of digital diagnostic tests?
7. Are relevant health professionals aware of these policies/strategies/plans? Where/how do they get the information?

Regulation

8. Can you describe the diagnostic pathway for the specific infectious disease area of focus described? Is this the same across all levels of health care delivery i.e. tertiary to community levels? If not, why?

9. Are there any specific regulations that exist for digital diagnostics tests?

10. Could you take us through the steps for introducing a new digital diagnostic test/medical device? *(Probe for enablers and barriers, approval processes, regulations for production, supply, administration and disposal of used materials)*

11. What mechanisms are in place to ensure that diagnostic tools used in the country meet international quality and safety standards? (Probe if *evidence requirements are applicable and appropriate for context, fake tests)*

12. How do these policies aim to balance innovation with patient safety and data privacy concerns?

Financing

13. How are diagnostic tests currently funded? Are existing funding mechanisms appropriate to serve populations requiring these tests? Please explain the answer.
    a. Are there existing funding/health technology assessment requirements for digital diagnostic tests?
14. Is it clear how new digital diagnostics tests for febrile illnesses can be funded?

15. What strategies or initiatives are being considered to overcome potential barriers to widespread implementation, such as reimbursement challenges or interoperability issues?

Identified need

5. What are your perceptions of new digital diagnostic tools for febrile illnesses, and how do they compare to traditional diagnostic methods? *Probe perception of user-friendliness, cost, quality, safety, and efficacy of new tests*

6. Given what you have described about existing tools, what is your perception of integrating a new digital diagnostic in Ghana's (or sub-Saharan African) health system?

7. What are the enablers/opportunities for optimal delivery and use of digital diagnostic tests across different levels of care?

8. What are the barriers/ challenges you anticipate, either related to regulatory issues, workforce capacity, or other factors when it comes to implementing these tests?

    a. How do you recommend these are mitigated?

Service provision

9. At what level and with which healthcare professionals are/should be involved in administering digital diagnostic tests?

10. How might the country ensure sufficient workforce capacity to meet current and future demand for digital diagnostic tests? (*Probe skills to use digital devices, capacity for maintenance and repair, spare parts etc*)

11. What determines/defines criteria for integration of digital diagnostics into the existing healthcare infrastructure and workflow?

    a. Do these criteria address access issues for different categories/levels of health care e.g. different levels (tertiary to community) and types (e.g. urban, peri-urban/urban slums and rural)

12. In this country context, what are the appropriate use cases or scenarios where digital diagnostics can be used?

    a. How would you prioritize these use cases?

    b. Do you think this would be the same across the health system levels described previously? Why or why not?

Health information

13. Please describe current data collection methods for diagnostic tests in the area of focus.
14. Do these methods appropriately capture data on access, efficacy, safety etc of diagnostic tests?
15. How is evidence generated incorporated into health system planning? Who is involved in the process?

Thank you for your time and valuable insights. If you have any additional thoughts, suggestions, or recommendations, please feel free to share them.
