# Supplementary material for: Health systems readiness for integration of point-of-care digital diagnostic tools for acute febrile illness in Ghana: a mixed-methods study protocol
Source: BMJ Public Health. 2026 Jul 14;4(3):e003704. doi: 10.1136/bmjph-2025-003704 (PMC13374460; doi:10.1136/bmjph-2025-003704)
Supplement: online supplemental file 3 [file bmjph-4-3-s003.pdf]

An assessment of readiness, integration and uptake of point of care (POC) digital diagnostic tools for febrile illness in Sub-Saharan Africa

Dear Health Policy and Systems Expert,

I am a PhD candidate in the Digital Diagnostic Network for Africa, at the University of Ghana. This survey is part of a study which aims to understand if health systems across sub-Saharan Africa are currently prepared for integration and use of point-of-care (POC) digital diagnostic tests i.e. tests that can be performed by a patient's bedside and provide actionable information at the location and time of care.

I would appreciate your participation in this research which involves completing this anonymous online survey. Before you commence the survey, here is some information about why this is being carried out.

What is the purpose of this research?

As part of a broader multi-country evaluation of new digital diagnostics in Africa, this research specifically aims to understand the policy and governance environments surrounding the integration of digital diagnostics within African health systems.

Why have I been invited to take part?

You have received this invitation because you are a health systems or policy expert working in Sub-saharan Africa. Your distinct viewpoint will offer invaluable insights into the readiness of African healthcare systems for point of care (POC) digital diagnostic tests.

What can I expect?

You will be asked to complete an anonymous survey with closed-ended questions in a Likert scale answer format. The survey will take about 15 minutes.

By submitting responses, you are providing consent for the data to be used in the study.

What are the benefits of participating in this survey?

By providing your expert opinions on the subject, you will contribute to a better understanding of the health system requirements to integrate digital diagnostic tests in a sub-Saharan country context and generate the evidence needed to inform the sustainable implementation of digital diagnostics.

Thank you for taking the time to read this information and for your valuable time on this evaluation!

If you have any questions or would like further information, please contact me Shola Dele-Olowu at skdele-olowu@st.ug.edu.gh or sholamole@gmail

Please complete the survey below.

Age	________________________________
Gender	◯ Male<br>◯ Female
How many years have you worked in the healthcare sector?	◯ Less than 1 year<br>◯ 1-5 years<br>◯ 6-10 years<br>◯ 11-15 years<br>◯ 16+ years
Town/ Country of facility/institution	________________________________

Current occupation	◯ Doctor<br>◯ Nurse<br>◯ Lab Technician<br>◯ Healthcare Administrator/Manager<br>◯ Other
Other specify	______________________________
Type of Healthcare Facility/Institution where you currently work	◯ Government<br>◯ NGO<br>◯ Academia<br>◯ Other
Specify name of facility/institution	______________________________
other specify	______________________________
How familiar are you with digital technology and devices in healthcare (i.e. electronic tools, systems, devices and resources that generate, store, or process data)?	◯ Very familiar<br>◯ Somewhat familiar<br>◯ Not very familiar<br>◯ Not familiar at all
Are you involved in decision-making that involves the use of digital technology in healthcare?	◯ Yes, directly<br>◯ Yes, indirectly<br>◯ No<br>◯ Other
Other specify	______________________________

Section B: To understand the existing policy and health system landscape for diagnosis of infectious diseases in your country

Are you aware of any existing national or regional policies in your country/ African country of support that guide the use of diagnostics tests?	◯ Yes<br>◯ No<br>◯ I don't know
Are there mechanisms in place to periodically evaluate and adapt existing policies related to diagnostic systems for infectious diseases?	◯ Yes<br>◯ No<br>◯ Unsure
Are there policies in place to ensure the quality and accuracy of diagnostic tests used for infectious diseases?	◯ Yes<br>◯ No<br>◯ I don't know

What gaps exist within the existing policy framework for infectious disease diagnostics?

- ☐ Limited access to advanced diagnostic technologies
- ☐ Challenges in integrating diagnostics into existing healthcare system workflows
- ☐ Insufficient funding for research and development
- ☐ Lack of standardized diagnostic protocols
- ☐ Inadequate inclusion of community perspectives in policy development
- ☐ Lack of coordination between public health agencies
- ☐ Focus on point-of-care diagnostics
- ☐ No clear guidelines for new diagnostic technologies
- ☐ Others

Other specify

____

How do you perceive the effectiveness of the existing policy framework for infectious disease diagnostics?

- ◯ Very effective
- ◯ Somewhat effective
- ◯ Somewhat ineffective
- ◯ Very ineffective
- ◯ Do not know

b. Assess strengths, limitations and gaps in the existing policy landscape for diagnostics of infectious diseases using the WHO REASSURED criteria

What do you believe are the primary challenges affecting delivery of diagnostic tests in your country? (Select all that apply)

- ☐ Affordability
- ☐ Availability
- ☐ Lack of healthcare infrastructure
- ☐ Insufficient healthcare workforce
- ☐ Inadequate medical supplies
- ☐ Access
- ☐ Quality of care
- ☐ Other

Other specify

____

Are there specific government incentives in place to promote the use and integration of digital diagnostic tools within the healthcare system?

- ◯ Yes
- ◯ No
- ◯ Don't know

Are there specific government initiatives aimed at promoting the integration of digital diagnostic tools within the healthcare system?

- ◯ Yes
- ◯ No
- ◯ Don't know

How do you get information about new digital diagnostic tests?

- ☐ Government
- ☐ UN agencies (WHO or Unicef)
- ☐ NGOs
- ☐ Other

If other, please specify

____

Are you aware of any new digital diagnostic point of care test (POCT) for febrile illnesses, such as advanced rapid diagnostic tests (RDTs)?

- ◯ Yes
- ◯ No
- ◯ Don't know

Is there information available about government policies or initiatives related to the integration of digital diagnostic tools in the healthcare system?	◯ Yes<br>◯ No<br>◯ Don't know
How do you perceive the acceptability of a new diagnostic test for malaria within the healthcare system?	◯ Highly acceptable<br>◯ Moderately acceptable<br>◯ Neutral<br>◯ Slightly acceptable<br>◯ Not acceptable
How would you rate the feasibility of integrating a new diagnostic test into existing healthcare practices?	◯ Highly feasible<br>◯ Moderately feasible<br>◯ Neutral<br>◯ Limited feasibility<br>◯ Not feasible
Regarding infrastructure and training requirements, how do you perceive the preparedness of the healthcare system in your country for the successful integration of a new digital diagnostic test?	◯ Well-prepared<br>◯ Moderately prepared<br>◯ Neutral<br>◯ Limited preparedness<br>◯ Not prepared
What strategies should be prioritized to ensure the successful implementation of a new POC digital diagnostic test? (Select all that apply)	☐ Training and capacity-building<br>☐ Integration with existing health systems<br>☐ Quality assurance and control<br>☐ Data management and analytics<br>☐ Continuous monitoring and evaluation<br>☐ Others
Other specify	______________________________

Healthcare worker's questionnaire for assessing the readiness of Ghana's health system to integrate and utilize new digital diagnostic tools

I would appreciate your participation in this research which involves completing this anonymous online survey. Before you commence the survey, here is some information about why this is being carried out.

What is the purpose of this research?

As part of a broader multi-country evaluation of new digital diagnostics in Africa, this research specifically aims to understand the policy and governance environments surrounding the integration of digital diagnostics within African health systems.

Why have I been invited to take part?

You have received this invitation because you are a health worker in Ghana. Your unique viewpoint will offer invaluable insights into the readiness of Ghana's healthcare system to integrate point-of-care (POC) digital diagnostic tests.

What can I expect?

You will be asked to complete an anonymous survey with closed-ended questions in a Likert scale answer format. The survey will take about 15 minutes.

By submitting responses, you are providing consent for the data to be used in the study.

What are the benefits of participating in this survey?

By providing your expert opinions on the subject, you will contribute to a better understanding of the health system requirements to integrate digital diagnostic tests in Ghana and generate the evidence needed to inform the sustainable implementation of digital diagnostics.

Thank you for taking the time to read this information and for your valuable time on this evaluation!

If you have any questions or would like further information, please contact me Shola Dele-Olowu at skdele-olowu@st.ug.edu.gh or sholamole@gmail

Please complete the survey below.

Participant ID	__________________________ (Please enter ID to begin with facility code e.g R = Ridge, and participant number e.g 01)
Age	__________________________
Gender	◯ Male<br>◯ Female
Current Educational level	◯ Primary school<br>◯ Secondary school<br>◯ Tertiary or Higher Education<br>◯ Vocational/Technical<br>◯ Other
Other specify	__________________________

Occupation	◯ Doctor<br>◯ Nurse<br>◯ Lab Technician<br>◯ Healthcare Administrator/Manager<br>◯ Other
Other specify	__________________________
How many years have you worked in the healthcare sector?	◯ Less than 1 year<br>◯ 1-5 years<br>◯ 6-10 years<br>◯ 11-15 years<br>◯ 16+ years
Type of Healthcare Facility/Institution where you currently work	◯ Hospital<br>◯ Clinic<br>◯ Health Center<br>◯ Laboratory<br>◯ Other
other specify	__________________________
Place of institution	◯ Urban<br>◯ Rural<br>◯ Suburban
People who work in this facility are confident in their capacity and skills to use diagnostic tests for febrile illness effectively	◯ Disagree<br>◯ Somewhat Disagree<br>◯ Neither agree nor disagree<br>◯ Somewhat agree<br>◯ Agree
People who work in this facility are aware of any new digital diagnostic POCT for febrile illnesses, such as advanced rapid diagnostic tests (RDTs)	◯ Disagree<br>◯ Somewhat Disagree<br>◯ Neither agree nor disagree<br>◯ Somewhat agree<br>◯ Agree
People who work in this facility feel confident they can contribute to using digital technology and devices in healthcare (i.e. electronic tools, systems, devices, and resources that generate, store, or process data such as smartphones	◯ Disagree<br>◯ Somewhat Disagree<br>◯ Neither agree nor disagree<br>◯ Somewhat agree<br>◯ Agree
People who work in this facility are convinced the introduction of new digital diagnostics tools is necessary in Ghana's healthcare system	◯ Disagree<br>◯ Somewhat Disagree<br>◯ Neither agree nor disagree<br>◯ Somewhat agree<br>◯ Agree
People who work in this facility believe the implementation of new digital diagnostics tools will improve the accuracy and speed of diagnosis in Ghana's healthcare system	◯ Disagree<br>◯ Somewhat Disagree<br>◯ Neither agree nor disagree<br>◯ Somewhat agree<br>◯ Agree

People who work in this facility believe that the successful integration of digital diagnostic tools will ultimately benefit both healthcare providers and patients	◯ Disagree<br>◯ Somewhat Disagree<br>◯ Neither agree nor disagree<br>◯ Somewhat agree<br>◯ Agree
People who work in this facility are motivated to adopt and implement the integration of new digital diagnosis tools in their health facilities	◯ Disagree<br>◯ Somewhat Disagree<br>◯ Neither agree nor disagree<br>◯ Somewhat agree<br>◯ Agree
People who work in this facility are committed to implementing new digital diagnosis tools in their health facilities	◯ Disagree<br>◯ Somewhat Disagree<br>◯ Neither agree nor disagree<br>◯ Somewhat agree<br>◯ Agree
People who work in this facility believe the facility has the necessary resources needed to effectively implement new diagnosis tools	◯ Disagree<br>◯ Somewhat Disagree<br>◯ Neither agree nor disagree<br>◯ Somewhat agree<br>◯ Agree
People who work in this facility feel confident that they can handle the challenges and disruptions that might arise from integrating new digital diagnosis tools in the facility	◯ Disagree<br>◯ Somewhat Disagree<br>◯ Neither agree nor disagree<br>◯ Somewhat agree<br>◯ Agree
People who work in this facility feel that the challenges and disruptions associated with integrating digital diagnostic tools is manageable within their current staffing levels	◯ Disagree<br>◯ Somewhat Disagree<br>◯ Neither agree nor disagree<br>◯ Somewhat agree<br>◯ Agree
People who work in this facility feel confident that they can coordinate tasks so that implementation of new digital diagnosis tools goes smoothly	◯ Disagree<br>◯ Somewhat Disagree<br>◯ Neither agree nor disagree<br>◯ Somewhat agree<br>◯ Agree
People who work in this facility feel confident that they can be trained to implement and use advanced digital diagnosis tools	◯ Disagree<br>◯ Somewhat Disagree<br>◯ Neither agree nor disagree<br>◯ Somewhat agree<br>◯ Agree
People who work in this health facility feel motivated to actively participate in the implementation process of digital diagnostic tools within the facility	◯ Disagree<br>◯ Somewhat Disagree<br>◯ Neither agree nor disagree<br>◯ Somewhat agree<br>◯ Agree
People who work in this facility believe the facility will do whatever it takes to make integrating new digital diagnosis tools into service delivery processes successful	◯ Disagree<br>◯ Somewhat Disagree<br>◯ Neither agree nor disagree<br>◯ Somewhat agree<br>◯ Agree

People who work in this facility believe the facility and government will provide the necessary requirements and support for the implementation of digital diagnosis tools into service delivery processes successful	◯ Disagree<br>◯ Somewhat Disagree<br>◯ Neither agree nor disagree<br>◯ Somewhat agree<br>◯ Agree
People who work in this facility feel confident the facility can get people invested in adopting and implementing new digital diagnosis tools in the facility	◯ Disagree<br>◯ Somewhat Disagree<br>◯ Neither agree nor disagree<br>◯ Somewhat agree<br>◯ Agree
People who in this facility feel confident that they can manage the politics of new digital diagnosis tools in the facility	◯ Disagree<br>◯ Somewhat Disagree<br>◯ Neither agree nor disagree<br>◯ Somewhat agree<br>◯ Agree
